# Correction to Crystal Structure of Poly(7-heptalactone)

**DOI:** 10.1021/acs.macromol.3c01301

**Published:** 2023-07-17

**Authors:** Anna Malafronte, Miriam Scoti, Maria Rosaria Caputo, Bo Li, Rachel K. O’ Reilly, Andrew P. Dove, Alejandro J. Müller, Claudio De Rosa

In our recent publication,^1^ we have reported the resolution of the crystal structure of a new polyester, the poly(7-heptalactone), which has been synthesized by ring-opening polymerization of η-heptalactone.

We have realized that in several parts of the paper and in the Supporting Information the description of the method of synthesis of the sample of poly(7-heptalactone) used for the structural characterization and resolution of the crystal structure is not correct. In fact, we wrote that “poly(7-heptalactone) (PHL) was synthesized by a combination of ring-opening polymerization of η-heptalactone (Scheme 1) and reversible addition–fragmentation chain transfer (RAFT) polymerization, using diphenyl phosphate (DPP) as catalyst and 4-cyano-4-(((ethylthio)carbonothioyl)thio) pentanoic acid as initiator and chain transfer agent”. The sample has been, instead, synthesized, as described in our previous paper,^2^ by ring-opening polymerization of η-heptalactone using diphenyl phosphate as catalyst and 4-cyano-4-(((ethylthio)carbonothioyl)thio)pentanoic acid as initiator.

The following sentences in our paper^1^ and the corresponding Supporting Information should be corrected as follows:

Abstract, lines 2–4. The sentence “PHL has been synthesized by a combination of ring-opening polymerization of η-heptalactone and reversible addition–fragmentation chain transfer polymerization using diphenyl phosphate as catalyst.” should be replaced with “PHL has been synthesized by ring-opening polymerization of η-heptalactone using diphenyl phosphate as catalyst.”

Introduction, page 4154, left column, lines 23–27. The sentence “In our recent previous report,^40^ PHL of low polydispersity of the molecular mass has been synthesized by ring-opening polymerization of η-heptalactone combined with reversible addition–fragmentation chain transfer (RAFT) polymerization using diphenyl phosphate as catalyst.” should be replaced with “In our recent previous report,^40^ PHL of low polydispersity of the molecular mass has been synthesized by ring-opening polymerization of η-heptalactone using diphenyl phosphate as catalyst.”

Experimental section, page 4154, left column, lines 1–5. The sentence “PHL was synthesized as described in our previous report,^40^ by a combination of ROP of η-heptalactone (Scheme 1) and reversible addition–fragmentation chain transfer (RAFT) polymerization, using diphenyl phosphate (DPP) as catalyst and 4-cyano-4-(((ethylthio)carbonothioyl)thio) pentanoic acid as initiator and chain transfer agent...” should be replaced with “PHL was synthesized as described in our previous report,^40^ by ROP of η-heptalactone (Scheme 1) using diphenyl phosphate (DPP) as catalyst and 4-cyano-4-(((ethylthio)carbonothioyl)thio) pentanoic acid as initiator”.

Supporting Information, page S2. The sentence “Poly(7-heptalactone) (PHL) was synthesized as described in our previous report,^S2^ by a combination of ring-opening polymerization (ROP) of η-heptalactone (Figure S2) and reversible addition–fragmentation chain transfer (RAFT) polymerization, using diphenyl phosphate (DPP) as catalyst and 4-cyano-4-(((ethylthio)carbonothioyl)thio) pentanoic acid as initiator and chain transfer agent...” should be replaced with “Poly(7-heptalactone) (PHL) was synthesized as described in our previous report,^S2^ by ring-opening polymerization (ROP) of η-heptalactone (Figure S2) using diphenyl phosphate (DPP) as catalyst and 4-cyano-4-(((ethylthio)carbonothioyl)thio) pentanoic acid as initiator”.

A corrected version of the Supporting Information is attached.

## Supporting Information Available

Correction to experimental details of synthesis; molecular and structural characterization by ^1^H NMR, ^13^C NMR, SEC, X-ray diffraction and DSC; ^1^H NMR and ^13^C NMR spectra and SEC curve of the synthesized sample of PHL; details of the preparation of single crystals, structure factors calculations, and crystal structure refinement (PDF)
