# Supplementary material for: Correction to Crystal Structure of Poly(7-heptalactone)
Source: Macromolecules. 2023 Jul 17;56(15):6173–4. doi: 10.1021/acs.macromol.3c01301 (PMC10413960; doi:10.1021/acs.macromol.3c01301)
Supplement: Supplementary file 1 — ma3c01301_si_001.pdf [file ma3c01301_si_001.pdf]

Supporting Information:

Crystal Structure of Poly(7-heptalactone)

Anna Malafronte,[†,*] *Miriam Scoti,*[†] *Maria Rosaria Caputo,*[‡] *Bo Li,*[§] *Rachel K. O' Reilly,*[§] *Andrew P. Dove,*[§] *Alejandro J. Müller,*[‡,‖] *Claudio De Rosa*[†,*]

[†] Dipartimento di Scienze Chimiche, Università degli Studi di Napoli Federico II, Complesso Monte S. Angelo, Via Cintia, I-80126, Napoli, Italy.

[‡] POLYMAT and Department of Polymers and Advanced Materials: Physics, Chemistry and Technology, Faculty of Chemistry, University of the Basque Country UPV/EHU, Paseo Manuel de Lardizábal, 3, 20018 Donostia-San Sebastián, Spain.

[§] University of Birmingham, Edgbaston, Birmingham, B152TT, United Kingdom.

[‖] IKERBASQUE, Basque Foundation for Science, Plaza Euskadi 5, 48009 Bilbao, Spain.

Correction to Supporting Information of the paper: *Macromolecules* **2023**, *56*, 4153−4162. DOI: 10.1021/acs.macromol.3c00710.

Experimental

Synthesis and characterization by NMR and Size Exclusion Chromatography.

The monomer η-heptalactone is not commercially available and was synthesized by Baeyer-Villiger oxidation of cycloheptanone with 3-chloroperbenzoic acid in CH_2Cl_2, according to the procedure reported in the literature (Figure S1).[S1]

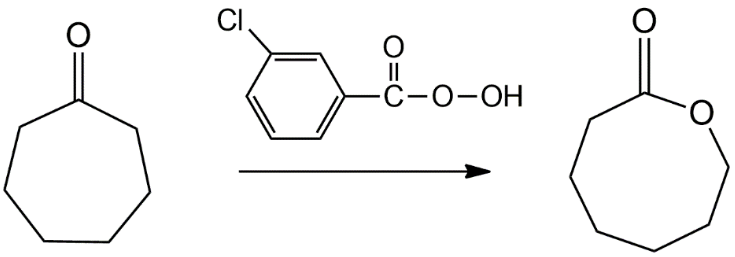

Figure S1. Scheme of the Baeyer-Villiger oxidation of cycloheptanone to synthesize η-heptalactone.

Poly(7-heptalactone) (PHL) was synthesized as described in our previous report,[S2] by ring-opening polymerization (ROP) of η-heptalactone (Figure S2) using diphenyl phosphate (DPP) as catalyst and 4-cyano-4-(((ethylthio)carbonothioyl)thio) pentanoic acid as initiator, according to the procedure also reported in Ref. S3 used for the synthesis of poly(ε-caprolactone) and different block copolymers based on poly(ε-caprolactone). Diphenyl phosphate was selected as catalyst due to its superior ability to provide polylactones with narrow dispersities.[S4]

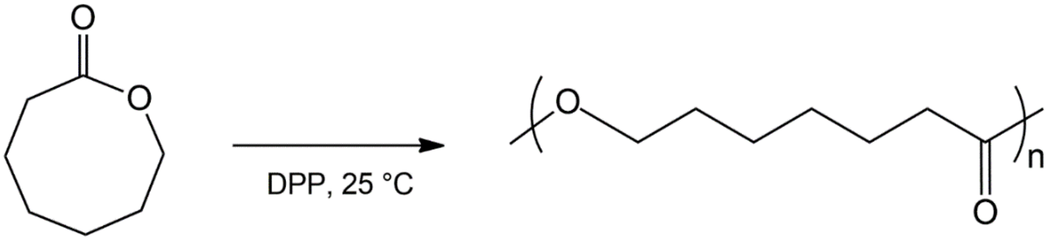

Figure 2. Scheme of the synthesis of the PHL by ring-opening polymerization of η-heptalactone catalyzed by DPP.

The microstructure of the PHL sample was analyzed with ^{1}H NMR and ^{13}C NMR spectroscopy. The average molecular mass was obtained by Size Exclusion Chromatography (SEC). The ^{1}H NMR and ^{13}C NMR spectra of the synthesized sample of PHL are reported in Figures S3 and S4, respectively. The size exclusion chromatogram is shown in Figure S5. The sample shows narrow distribution of molecular masses with $M_w/M_n \approx 1.15$ according with the synthetic strategy.

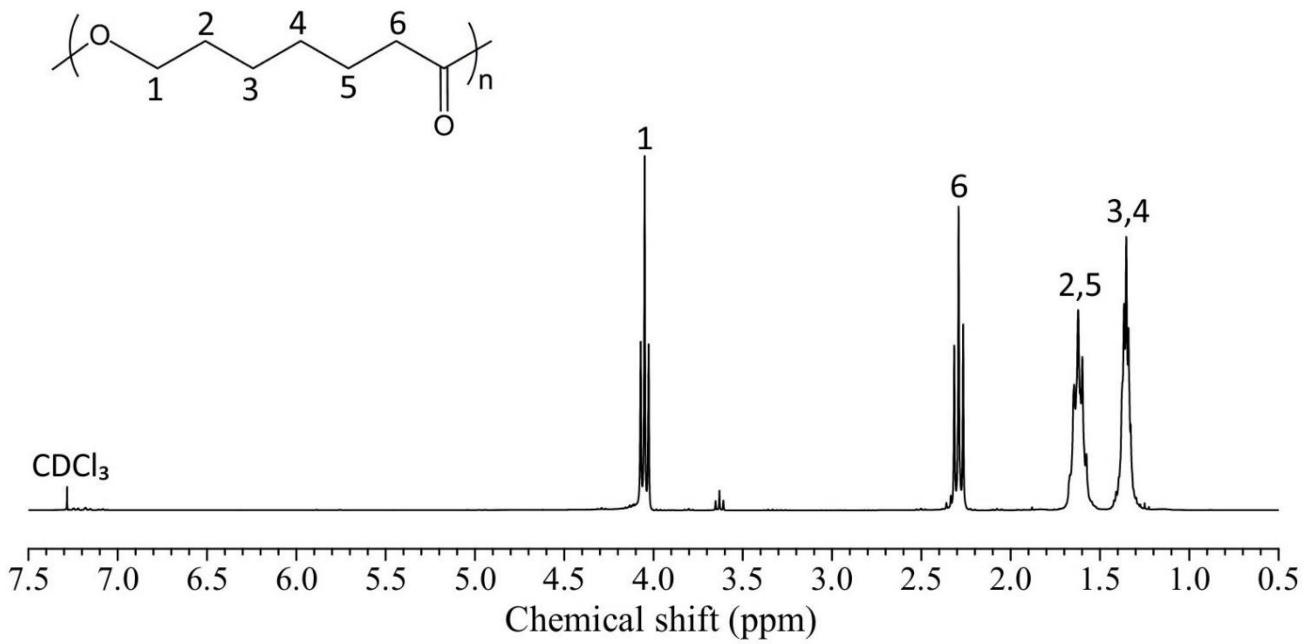

Figure S3. ^{1}H NMR spectrum of the synthesized sample of PHL in $CDCl_3$ solution. The assignment of the observed resonances to hydrogen atoms of the methylene groups shown in the scheme is indicated.

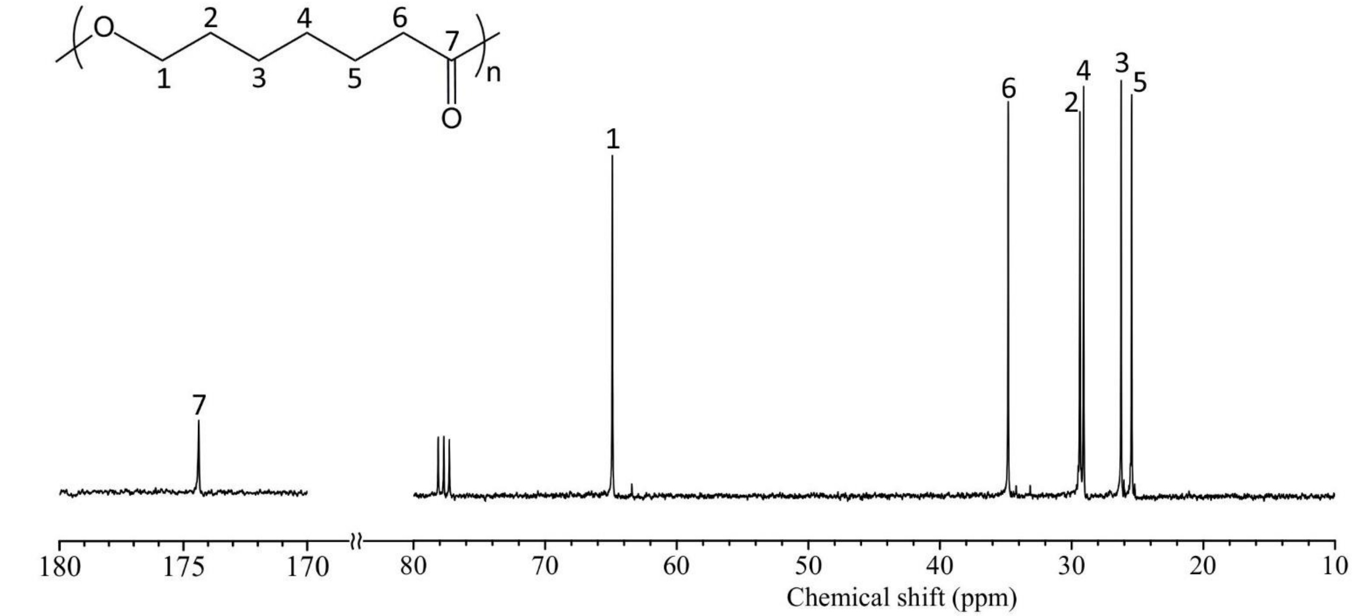

Figure S4. ^{13}C NMR spectrum of the synthesized sample of PHL in $CDCl_3$ solution. The assignment of the observed resonances to carbon atoms shown in the scheme is indicated.

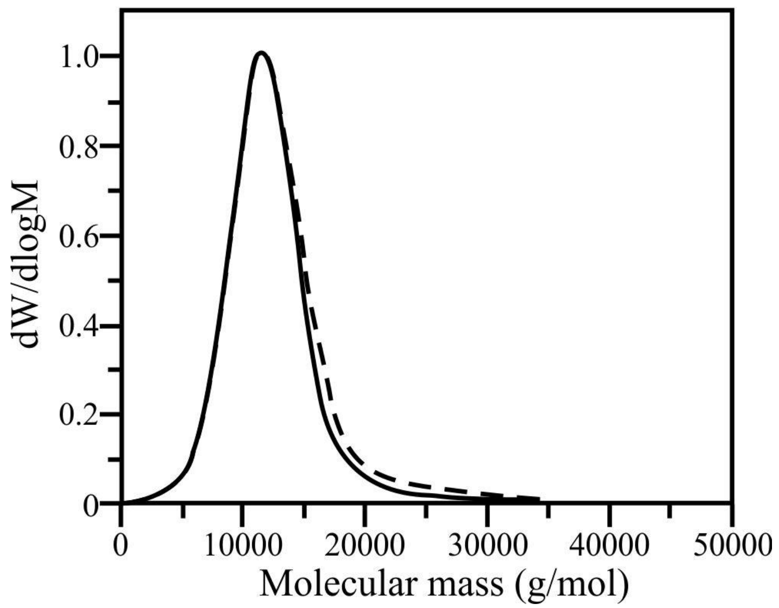

Figure S5. Ultraviolet (continuous curve) (λ = 309 nm) and refractive index (dashed curve) SEC chromatograms of PHL using $CHCl_3$ with 0.1% trifluoroethanol as an eluent with polystyrene (PS) standards.

Characterization by X-ray diffraction and DSC

Melt-crystallized sample was prepared by heating the as-synthesized sample at a temperature 30 - 40°C above the melting temperature between the plates of a press, and then cooling to room temperature by circulating of cold water inside the press plates (estimated cooling rate ≈ 20°C/min).

The melting and crystallization temperatures of PHL were obtained with the differential scanning calorimeter (DSC) Mettler Toledo DSC 3+, calibrated with indium, by performing scans in a flowing N_2 atmosphere at heating and cooling rate of 10 °C/min.

X-ray powder diffraction profiles of the as-prepared (precipitated from the polymerization solution) and melt-crystallized sample were obtained with Ni filtered Cu Kα radiation (λ = 1.5418 Å) by using an Empyrean diffractometer by Malvern Panalytical operating in the reflection geometry with continuous scans of the 2θ angle and scanning rate of 0.021 degree/s.

The degrees of crystallinity (x_c) were determined from the powder diffraction profiles by the ratio between the crystalline diffraction area (A_c) and the area of the whole diffraction profiles (A_t), x_c =

$(A_c/A_t)\times100$. The area of the crystalline diffraction A_c was evaluated by subtracting the area of the amorphous halo from the area of the whole diffraction profiles A_t.

Preparation and characterization of single crystals. A 0.012 wt% solution of PHL in 1-hexanol was prepared by dissolving the polymer (0.3 mg) into 3 mL of solvent. The solution was placed at 85 °C and maintained at this temperature for 1 h to completely dissolve the polymer. The solution was then slowly cooled down to 50 °C (estimated cooling rate ≈ 1.2 °C/min) and kept at this temperature for 21 h to allow crystallisation. Afterward, the solution was slowly cooled to room temperature (estimated cooling rate lower than 1 °C/min). Drops of crystal suspension were deposited on carbon-coated grids and allowed to dry before transmission electron microscopy (TEM) and electron diffraction (ED) analysis. Bright-field TEM images of crystals and ED patterns were acquired by using a FEI TECNAI G2 200 kV S-TWIN microscope (electron source with LaB_6 emitter) operating at 120 kV.

Structural analysis, energy calculation and crystal structure refinement.

Structural modeling, energy calculations, X-ray powder diffraction simulations and full-profile refinement were performed by using Accelrys BIOVIA Materials Studio (MS) software package. Energy calculations were performed by using the consistent-valence forcefield (CVFF).[S5] No van der Waals and electrostatic interactions for distances longer than 20 Å were taken into account.

The observed diffraction intensities (I_o) were evaluated from the experimental X-ray powder diffraction profile by measuring the area of the peaks after subtraction of a straight baseline approximating the background and of the amorphous contribution. The calculated diffraction intensities (I_c) for the final model of packing were calculated form the moduli of the calculated structure factors $F_c(hkl)$ of the hkl reflections as: $I_c = \Sigma|F_c(hkl)_i|^2 \times M_i \times LP$, where $F_c(hkl)_i$ is the structure factor and M_i the multiplicity factor of the hkl reflection i, and the sum is taken over all reflections i included in the 2θ range of the corresponding diffraction peak observed in the X-ray powder diffraction profile. For each hkl reflection at the Bragg angle 2θ, LP is the Lorentz-polarization factor for X-ray powder diffraction:

$LP = (1+\cos^2 2\theta)/(\sin^2\theta\cos\theta)$.

Anisotropic thermal factors were assumed. The values of the atomic scattering factors as given in Ref. S6 were used for the calculation of the structure factors. The observed intensities were scaled so that $\sum I_o = \sum I_c$.

The crystal structure was refined by Rietveld powder diffraction refinement by using the Rietveld program implemented in the Powder Refinement tool of MS package. The program is based on standard non-linear least squares algorithms employed in Rietveld refinement.[S7,S8] The agreement between the calculated and the experimental diffraction profiles was evaluated from the figures of merit of the best fit, defined as the weighted profile residual factor (R_{wp}) and the profile residual factor (R_p), calculated by equations 1 and 2, respectively:

$$R_{wp} = \left(\frac{\sum_i w_i \left(cI^{calc}(2\theta_i) - I^{exp}(2\theta_i) + I^{back}(2\theta_i) \right)^2}{\sum_i w_i \left(I^{exp}(2\theta_i) \right)^2} \right)^{1/2} \quad (1)$$

$$R_p = \frac{\sum_i |cI^{calc}(2\theta_i) - I^{exp}(2\theta_i) + I^{back}(2\theta_i)|}{\sum_i |I^{exp}(2\theta_i)|} \quad (2)$$

where $I^{exp}(2\theta_i)$ is the measured experimental diffraction intensity at the value $2\theta_i$ of the Bragg angle, $I^{back}(2\theta_i)$ is the background intensity at the angle $2\theta_i$ and $I^{calc}(2\theta_i)$ is the calculated diffraction intensity at the angle $2\theta_i$ without the background contribution, $w_i = 1/I^{exp}(2\theta_i)$ is the weight of each observed reflection assumed to be the reciprocal of its intensity and c is a constant scaling factor optimized to obtain the lowest value of R_{wp} and R_p. The sums in Equations 1 and 2 extend over the defined range of the values of the Bragg angle 2θ.

References

S1) Van Der Mee, L.; Helmich, F.; De Bruijn, R.; Vekemans, J. A. J. M.; Palmans, A. R. A.; Meijer, E. W. Investigation of Lipase-Catalyzed Ring-Opening Polymerizations of Lactones with Various Ring Sizes: Kinetic Evaluation. *Macromolecules* **2006**, *39*, 5021–5027.

S2) Caputo, M. R.; Olmos, A.; Li, B.; Olmedo-Martínez, J. L.; Malafronte, A.; De Rosa, C.; Sardon, H.; O' Reilly, R. K.; Dove, A. P.; Müller, A. J. Synthesis, morphology and crystallization kinetics of Polyheptalactone (PHL). *Biomacromolecules* **2023**, *24*, 3256-3267.

S3) Arno, M. C.; Inam, M.; Coe, Z.; Cambridge, G.; Macdougall, L. J.; Keogh, R.; Dove, A. P.; O'Reilly, R. K. Precision Epitaxy for Aqueous 1D and 2D Poly (ε-Caprolactone) Assemblies. *J. Am. Chem. Soc.* **2017**, *139*, 16980.

S4) Ottou, W. N.; Sardon, H.; Mecerreyes, D.; Vignolle, J.; Taton, D. Update and Challenges in Organo-Mediated Polymerization Reactions. *Prog. Polym. Sci.* **2016**, *56*, 64–115.

S5) Dauber-Osguthorpe, P.; Roberts, V. A.; Osguthorpe, D. J.; Wolff, J.; Genest, M.; Hagler, A. T. Structure and energetics of ligand binding to proteins: E. coli dihydrofolate reductase-trimethoprim, a drug-receptor system *Proteins: Struct. Funct. Genet.* **1988**, *4*, 31-47.

S6) International Table for X-ray crystallography, Vol. B: Reciprocal Space, Shmueli U. ed., Springer, Dordrecht, 2008.

S7) Rietveld, H. M. A profile refinement method for nuclear and magnetic structures *J. Appl. Cryst.* **1969**, *2*, 65-71.

S8) Young, R. A. The Rietveld Method, IUCr Monographies of Crystallography, 5, Oxford University Press, Oxford **1993**.
